# The Effects of Physical Running on Dendritic Spines and Amyloid-beta Pathology in 3xTg-AD Male Mice

**DOI:** 10.14336/AD.2022.0110

**Published:** 2022-07-11

**Authors:** Benke Xu, Yun He, Lian Liu, Guosheng Ye, Lulu Chen, Qingning Wang, Michael Chen, Yuncai Chen, Dahong Long

**Affiliations:** ^1^Department of Human Anatomy, School of Basic Medical Sciences, Yangtze University, Hubei 434023, China.; ^2^Department of Pharmacology, School of Basic Medical Sciences, Yangtze University, Hubei 434023, China.; ^3^Key Lab of Neuroscience, School of Basic Medical Sciences, Guangzhou Medical University, Guangzhou 511436, China.; ^4^University of California, Los Angeles, CA 90095, USA.; ^5^Department of Pediatrics, University of California, Irvine, CA 92697, USA.

**Keywords:** dendritic spines, synapses, amyloid, memory, Alzheimer’s disease, physical training

## Abstract

Memory loss is the key symptom of Alzheimer's disease (AD). As successful drug treatments have not yet been identified, non-pharmaceutical interventions such as physical exercise and training have been employed to improve the memory function of people with dementia. We investigated the effect of prolonged physical running on hippocampal-dependent spatial memory and its underlying mechanisms using a well-established rodent model of AD. 3xTg-AD transgenic mice and non-transgenic mice were subjected to voluntary wheel running for 5 months (1 hour per day, 5 days per week), followed by spatial memory testing. After the behavioral testing, dendritic spines, synapses, and synaptic proteins as well as amyloid-beta (Aβ) pathology were analyzed in the dorsal hippocampi. Running improved hippocampal-dependent spatial memory in 3xTg-AD mice. This running strategy prevented both thin and mushroom-type spines on CA1 pyramidal cells in 3xTg-AD mice, whereas the effects of running in non-transgenic mice were limited to thin spines. The enormous effects of running on spines were accompanied by an increased number of synapses and upregulated expression of synaptic proteins. Notably, running downregulated the processing of amyloid precursor protein, decreasing intracellular APP expression and extracellular Aβ accumulation, and spatial memory performance correlated with levels of Aβ peptides Aβ_1-40_ and Aβ_1-42_. These data suggest that prolonged running may improve memory in preclinical AD via slowing down the amyloid pathology and preventing the loss of synaptic contacts.

The progressive loss of memory in patients with Alzheimer's disease (AD) is accompanied by several primary pathological features including an extensive loss of synaptic connections and abnormal deposits of amyloid plaques in the brain [1,2]. AD is categorized into early-onset and late-onset forms (familial AD and sporadic AD, respectively), and the latter accounts for more than 98% of AD cases. While the cause of AD is not yet fully understood, it is generally believed that early-onset Alzheimer’s is a result of genetic mutation and that late-onset Alzheimer’s arises from a combination of genetic, environmental, and lifestyle factors [1,3]. Despite current intensive research, successful drug treatments have yet to be identified. Because AD is complex and the actual underlying cause of this disease is poorly defined [1,2,4-6], non-pharmaceutical interventions that may help reduce the risk of cognitive decline and delay the onset of AD are worthy of attention.

Physical exercise is a simple intervention that remarkably enhances cognition and has been recognized as an effective strategy to improve the learning and memory functions of aged people with or without cognitive decline [7-15]. Although it is not yet certain whether exercise can alleviate and/or delay memory deficits in patients who have been diagnosed with AD [16], data from clinical trials have shown that regular moderate physical exercise has beneficial effects on dementia patients, particularly those with mild dementia [12,14,17,18]. It should be noted that the effects of physical exercise on patients with AD are largely dependent on the type, frequency, and intensity of exercise applied, as reported in older people [19]. How does physical exercise benefit learning and memory functions while reducing the risk of dementia? An increasing body of evidence suggests that physical exercise can lead to structural and functional changes in brain regions important for learning and memory, such as the hippocampus [20-25]. Studies in aged animals [26-28] and in animal models of neurodegenerative diseases [ *e.g.*, 29, 30] have shown that voluntary exercise and activity can promote neurogenesis and cell proliferation as well as enhance synaptic plasticity. We have reported that voluntary wheel running started at an older age in mice (16 months old) can prevent aging-related selective loss of presynaptic inputs and postsynaptic dendritic spines in the hippocampus [31,32]. Importantly, the degree of running-provoked increase of dendritic spines, particularly thin spines bearing small synapses, positively correlates with spatial memory performance in individual animals [31,33]. This suggests that preserved synaptic contacts contribute critically to the improved memory function of these animals. The early stages of AD pathogenesis are believed to occur at dendritic spines and synapses, because loss of these structures best correlates with poor memory performance [34,35]. Furthermore, soluble oligomeric forms of amyloid-beta (Aβ) and amyloid precursor protein (APP) can reduce synaptic transmission and deteriorate synapses, disrupting cognitive function [36-38]. While improved memory function via physical running has been linked to the conservation of pre- and post-synaptic contacts in normal aging mice [31,32], it is unknown whether voluntary physical running has beneficial effects on the dendritic structure or Aβ-pathology, which is highly associated with synaptic plasticity and function in AD animal models.

To explore whether voluntary exercise prevents and/or postpones AD progression, we have investigated the impacts of voluntary running on spatial memory, dendritic spines, synaptic contacts, and Aβ-pathology in 3xTg-AD transgenic mice, a well-established rodent model of AD. We have found that voluntary running for 5 months improves the spatial memory of 3xTg-AD mice, measured via a novel object location task and a water maze task. When compared with 3xTg-AD sedentary mice, increased numbers of spines and synapses are apparent in area CA1 of the dorsal hippocampi of 3xTg-AD running mice. This is accompanied by a higher expression of the synaptic proteins postsynaptic density protein-95 (PSD-95), AMPA glutamate receptor subunit GluR1, and presynaptic vesicle protein synaptophysin. Furthermore, running reduces Ab peptides and oligomers by modulating APP processing via the downregulation of BACE1 steady state levels. A reduced accumulation of Aβ peptides/oligomers is also apparent in the radiatum of area CA1. Because the levels of Ab peptides in hippocampal CA1 correlate with spatial memory performance, these data suggest that physical running can postpone amyloid pathogenesis and prevent age-related loss of spines and synaptic proteins in hippocampal CA1 of 3xTg-AD mice, contributing to improved spatial memory.

## MATERIALS AND METHODS

### Animals

Homozygous 3xTg-AD transgenic mice (3xTg-AD) containing *Psen1, APPSwe*, and *tauP301L* mutations and non-transgenic controls (Ntg) with a B6;129 genetic background were generated from breeders purchased from the Jackson Laboratories (Stock No. 004807, Bar Harbor, ME, USA). The mice were group-housed in a quiet facility and maintained on a 12-hour light/dark cycle (lights on at 7 am), with *ad libitum* access to food and water. Experiment procedures were carried out according to the National Institutes of Health Guide for Care and Use of Laboratory Animals and approved by the Bioethics Committees of the Yangtze University and Guangzhou Medical University. Male mice were subjected to a 5-month voluntary wheel running period initiated at 8 weeks of age and compared with their age-matched sedentary controls. Two cohorts of mice were used, and each contained 3xTg-AD runner, 3xTg-AD sedentary control, Ntg runner, and Ntg sedentary control. At the end of running, one cohort of mice was subjected to a relatively ‘stress-free’ object location task, followed by analyses of spines and synapses, while the other was subjected to a classic water maze task, followed by measures of Aβ pathology.

### Physical training

Physical training was performed as described previously [31,32]. Briefly, mice were individually placed in vertically revolving activity wheels (16 cm in diameter and 5 cm in depth) and trained for 2 weeks (6-10 pm), followed by 5 months of wheel running (7-10 pm). During the training session, mice learned to run 10 min on the first day, and running time progressively increased by 10 min per day until they ran 1 h per day. After the training, mice that had learned to run voluntarily in wheels were chosen and assigned to running and sedentary groups. Mice in the running group continued to run for 1 h per day during the dark cycle for 5 days a week. The sedentary controls were placed in immobile running wheels for 1 h per day. Because the wheel running is voluntary, we set a criterion for effective training in the runners such that the heart-to-body weight ratio is at least 2 standard deviations (SD) above the mean in sedentary controls (Mean + 2 SD) [31,32]. 12 in 18 Ntg runners and 13 in 18 3xTg-AD runners in one cohort of mice fulfilled the standard, while 10 in 16 Ntg runners and 11 in 16 3xTg-AD runners in the other cohort were selected for data analyses.

### Behavioral tests of memory function

A novel object location task was employed to test hippocampal-dependent spatial memory [33]. Prior to training, mice were handled for 2 min and habituated to an experimental apparatus for 10 min per day for 5 days in the absence of objects. In the training phase, two identical objects were presented for 10 min of exploration. One day later, object exploration was tested for 5 min. The training and testing were performed without knowledge of groups. A video tracking system (EthoVision, Noldus, the Netherlands) was used to record both training and testing phases. Exploration was scored when a mouse's head was oriented toward the object within 1 cm or when the nose was touching the object. Exploration times of the object in familiar and novel locations were recorded and expressed as a novel vs. familiar ratio. Total exploration times were calculated, and the novel/familiar ratio was used as an index of memory function.

The water maze test was conducted with the aid of a video tracking system (EthoVision) as described [31,32]. The maze consists of a circular tub (120 cm in diameter) and a transparent non-slip platform (9 cm in diameter) which was placed in a constant position for each set of trials. The platform was placed in the middle of a quadrant 1 cm below the surface of room temperature water. During a 5-day training period (3 trials per day), mice explored the platform and learned to associate its location to visual cues in the room. If mice could not locate the platform within 60 seconds, they were gently guided to it. One day after the last trial of day 5, probe trials (1 min each) in which the platform was removed were conducted. During the probe testing, mice were placed in the water facing the pool wall, the number of crossing the original location of the platform and the duration of stay in each quadrant were recorded.

### Tissue handling, Golgi staining, and immunostaining

At the end of the object location test, mice were anesthetized with sodium pentobarbital (80 mg/kg) and perfused via the aorta with 0.9% saline solution for 2 min to flush out blood. The brains were rapidly removed from the skull and blocked to two hemispheres. The left hemispheres were harvested for Golgi staining while the right hemispheres were collected for immunostaining.

Golgi staining was performed using a superGolgi kit (Bioenno Tech LLC, Santa Ana, CA). In brief, after 2 days of impregnation in the Golgi-Cox solution (22 ± 1°C, in the dark) provided, the solution was renewed and the impregnation was continued for another 7 days (22 ± 1°C), for a total of 9 days. The tissue blocks were sectioned coronally at 200 µm (Leica VT1200). Series sections from the dorsal hippocampus (1 in 3, 6-8 sections per brain, Bregma -1.06 mm to -2.54 mm) were mounted on gelatin-coated slides and subjected to staining (15 min, 22 ± 1°C) and post-staining (18 min, 22 ± 1°C) in a parallel manner. Sections were dehydrated in ethanol, cleared in xylene, and covered with Permount® mounting medium.

Immunostaining of postsynaptic density protein-95 (PSD-95) was performed on free-floating sections [33]. Briefly, brain tissues were fixed in 4% paraformaldehyde in 0.1 M phosphate buffer (PB) (pH7.4) overnight at 4°C, and fixed tissue blocks were cryoprotected in 30% sucrose in 0.1 M PB and sectioned coronally (20 µm) using a Leica cryostat. A series of sections (every sixth section from the dorsal hippocampus, Bregma -1.22 mm to -2.54 mm) was subjected to fluorescent immunostaining. The sections were incubated with mouse monoclonal anti-PSD-95 (1:2,000, MA1-25629, clone 7E3-1B8, Affinity BioReagents, Golden, CO) for 3 days at 4°C in a parallel manner. After PBS-T washing (3 × 5 min), antibody binding was visualized using anti-mouse IgG conjugated to Alexa Fluor 488 (1:200, Molecular Probes).

### Quantitative analyses of dendritic spines and synapses

Dendritic spines: Two approaches were applied to evaluate the number and density of dendritic spines in Golgi-stained sections. 1) Spines in area CA1 were counted with the aid of Stereo Investigator (MBF Bioscience, Williston, VT, USA) using the stereological fractionator method [39,40]. A series of sections (every third section) from the dorsal hippocampus was subjected to counting. Strata radiatum (SR) and lacunosum-moleculare (SLM) in area CA1 were defined using a 5× objective, and spines were counted using a 100×/1.4 objective. We used a counting frame of 25 × 25 μm, a sampling grid of 200 × 200 μm, a guard zone of 10 μm, and a disector height of 50 μm. 2) Spine density (number of spines per 20 µm of dendrite length) was calculated on individual CA1 pyramidal cells (3-4 cells per brain). Well-impregnated cells were captured with a Nikon E400 equipped with a CCD camera (DS-Fi3) and motorized stage and reconstructed using Imaris (v7.1.0) and Adobe Photoshop (v6). Cells in each group included equal representation of long- and short-shaft populations [33]. High-magnification images (100×/1.4 oil lens) permitted all spines of a given dendritic segment to be visualized. The sides of dendritic branches were carefully examined for vertical protrusions stretching upward and downward off the branch. Branches including basal dendrites in the oriens (SO), apical obliques in SR, and apical distal branches in SLM were analyzed. The apical trunk was not analyzed because some spines that extended vertically toward the observer were difficult to classify from an aerial perspective. Density of spines on each branch was separately analyzed with the aid of concentric circles at an interval of 20 μm. The data were grouped by animal. Spines were classified by size and shape as mushroom-type, thin, or stubby [33,41].

Synapses: The number of synapses represented by PSD-95 immunoreactive (ir) puncta were quantitatively analyzed as described [32,33]. 10 sections (90 µm interval each other) per animal were used, and z-stack images were taken from the regions of interest using a Zeiss 510 confocal microscope with a 63× objective. The region of interest was defined using a 4× objective and images were then captured using a 63×/0.8 objective. We used a counting frame of 25 × 25 µm, a sampling grid of 200 × 200 µm, a guard zone of 10 µm, and a disector height of 5 µm. The precision of the study was estimated by calculating the coefficient of error (CE). The CE value for each individual animal ranged between 0.02 and 0.05. Confocal three-dimensional image stacks were processed for iterative deconvolution at 99% confidence (Volocity 6.3). Counts (per 7,500 µm^3^) of labeled puncta from each section were averaged to obtain a value for each brain. Sections from four groups were processed concurrently and analyzed without knowledge of treatment group.

### Preparation of protein extracts and Western blot analyses of synaptic proteins

The dorsal hippocampus was dissected and area CA1 was further isolated. Dissected tissue was immediately frozen in dry ice and homogenized in T-PER Tissue Protein Extraction Reagent (ThermoFisher Scientific, Rockford, IL) (150 mg/ml) containing protease and phosphatase inhibitor cocktail (1:100, Sigma-Aldrich, St. Louis, MO). The sample was centrifuged at 100,000× g for 1 h. The pellet was re-suspended with 70% formic acid, followed by centrifugation at 100,000× g for another hour. Protein concentration in the supernatant was determined using the Bradford assay.

Equal amounts of protein (20 μg) were diluted in Laemmli buffer, separated on 4-12% Bis-Tris gel (Invitrogen, Carlsbad, CA), and transferred to nitrocellulose membranes. The membranes were blocked using 5% nonfat milk in 1× TBS-T overnight at 4°C, followed by incubation in primary antibodies overnight at 4°C. The antibodies included mouse anti-PSD95 (1:5,000, clone 7E3-1B8), rabbit anti-GluR1 (1:2,000; PC246, Calbiochem), and mouse anti-synaptophysin (1:10,000, Sigma). The membranes were washed in TBS-T (3 × 5 min) and incubated in the appropriate secondary antibody (anti-rabbit IgG or anti-mouse IgG conjugated to HRP) at a 1:10,000 dilution (Pierce Biotech) for 1 h at room temperature (RT). Membranes were then washed in TBS-T (3 × 5 min), and the blots were developed using SuperSignal (Thermo Scientific). Hippocampal extracts of individual mice of different groups were run concurrently on the same gel. Signal specificity was verified by pre-adsorbing primary antibodies with their respective antigens as well as by excluding the primary antibodies in the presence of the secondary antibodies. These treatments resulted in no immunoreactive bands.

### Analyses of Aβ peptides, oligomers, and APP secretases

ELISA of Aβ peptides: Aβ_1-38_, Aβ_1-40_, and Aβ_1-42_ were measured using the 6E10 Abeta Peptide Ultra-Sensitive Kits (K151FSE, K151FTE, and K151FUE, respectively, MSD, Rockville, MD, USA) [40]. T-PER soluble fractions from the dorsal hippocampus were loaded directly onto the ELISA plate, and the formic acid supernatants (insoluble fractions) were diluted 1:2 in neutralization buffer (1M Tris base and 0.5M NaH_2_PO_4_) prior to loading. After incubation in blocking solution, samples and standard peptides including Aβ_1-38_, Aβ_1-40_, and Aβ_1-42_ were added to the 96-well plate and incubated overnight (4°C), followed by washes in 1× Tris wash buffer (3 × 5 min). After a 1 h incubation in detection solution (RT), the plate was washed with 1× Tris buffer and read in a Sector Imager plate reader (MSD) immediately after addition of the 1× read buffer. The concentration of Aβ peptides was calculated with reference to the standard curves and expressed as picograms per milligram of proteins.

Dot-blot of Aβ oligomers and amyloid fibril: Equal amounts of protein (3 μg) were transferred to nitrocellulose membranes, and the membranes were blocked using 5% (w/v) nonfat milk in 1× Tris-buffered saline containing 0.2% Tween 20 (TBS-T, pH 7.5) for 1 h at RT. The membranes were then incubated overnight at 4°C with one of the following primary antibodies: rabbit anti-oligomer (A11) polyclonal (AHB0052, 1:1,000, ThermoFisher) or rabbit anti-amyloid fibrils OC (AB2286, 1:3,000, Sigma-Aldrich). A11 reacts with soluble AB40 oligomers, but not with soluble low molecular weight AB40 or AB40 fibrils. The membranes were washed in TBS-T (3 × 5 min) and incubated in goat anti-rabbit IgG secondary antibody (1:10,000; ThermoFisher) for 1 h at RT. The blots were developed using SuperSignal chemiluminescent substrates (ThermoFisher).

Western blot analyses of APP and secretases: Equal amounts of protein (20 μg) were diluted and transferred to nitrocellulose membranes as described above. The membranes were blocked using 5% nonfat milk in 1× TBS-T overnight at 4°C, followed by incubation in primary antibodies overnight at 4°C. The antibodies included rabbit anti-APP-CT20 for C99 and C83 (1:1,000, Calbiochem, San Diego, CA), rabbit anti-ADAM10 (1:1,000, MA5-32616, ThermoFisher), rabbit anti-ADAM17 (1:1,000, PA5-11572, ThermoFisher), rabbit anti-BACE1 (1:1,000, MA5-35126, ThermoFisher), and mouse anti-GAPDH (1:5,000, sc-47724, Santa Cruz Biotech, Santa Cruz, CA). The membranes were incubated in the appropriate secondary antibody, and the blots were developed using SuperSignal as described.

Aβ pathology on sections: Immunohistochemistry (IHC) and immunofluorescence (IF) were performed on free-floating hippocampal sections (20 µm) as described [32,33]. The matched sections from 3xTg-AD sedentary and running mice were incubated with primary antibodies for 2 days at 4°C. The following primary antibodies were used: mouse anti-APP (6E10) (1:1,000, cat # 803001, BioLegend, CA), rabbit anti-Aβ40 (1:1,000, AB5074P, Millipore, MA), rabbit anti-Aβ42 (1:1,000, AB5078P, Millipore), rabbit anti-amyloid fibrils OC (1:500, AB2286, Sigma-Aldrich), and mouse anti-GFAP (1:5,000, cat # 3402, Sigma-Aldrich). For IHC, after the PBS-T wash (3 × 5 min), the sections were incubated in biotinylated anti-mouse IgG (1:400, Vector) for 2 h at room temperature (RT) and avidin-biotin-peroxidase complex solution (ABC, 1:200, Vector) for 3 h at RT. The reaction product was visualized by incubating the sections for 10 min in 3,3’-diaminobenzidine (DAB) containing H_2_O_2_ (Bioenno Tech LLC, Santa Ana, CA). For IF, antibody binding was visualized using anti-mouse or anti-rabbit IgG conjugated to Alexa Fluor 488 or 568 (1:200, 2 h at RT, Molecular Probes). Combined anti-OC and anti-GFAP (1:500 and 1:5,000, respectively) were used for dual labeling IF.

Quantification: The coronal sections were derived from the dorsal hippocampus (Bregma -1.22 mm to -2.54 mm) of 3xTg-AD sedentary and running mice (n = 6 per group). Four matched sections per animal were selected for imaging and analysis. The optical density (OD) of immunoreactivity was calculated using ImageJ (v2) based on the average value from three fields containing the layers of interest. The OD of the corpus callosum on the same section was measured and served as the background [32]. The number of Aβ-immunoreactive plaques or granules in the radiatum (per 450 µm × 200 µm) was counted using Imaris (v9.2.1, Bitplane Inc.) and ImageJ (v2). Confocal images (20× and 63× objectives, Zeiss 510) were captured as described above. Counts of labeled granules from each section were averaged to obtain a value for each animal. Sections from two groups were analyzed without knowledge of treatment group. For the OC-ir granules, the number of granules in CA1 radiatum (per 450 µm × 200 µm) and area per granule were analyzed. The signal of individual granules inside of the selected cluster was kept in a consistent threshold mask during the analysis.

### Statistical analysis

Data were analyzed using Prism 9 (GraphPad Software Inc.) or SPSS 22.0 (SPSS Inc.). Analyses of variance (ANOVA), including two-way and three-way, were used to detect differences in the escape latencies, path lengths, and swimming speeds in the water maze task with groups (Ntg vs. 3xTg-AD, sedentary vs. running) and time (day) as factors, followed by Tukey’s or Bonferroni’s *post hoc* test. Two-way ANOVA was employed to compare the exploration times and novel/familiar ratios among groups. A one-sample *t*-test was also performed to distinguish whether exploration times of objects were significantly different from those predicted by chance. Spines, synapses, and synaptic proteins were analyzed by three-way or two-way ANOVA with groups and branch segment and/or subregion/subtype as factors, followed by Tukey’s or Bonferroni’s *post hoc* test. Comparison between two groups was performed using Student’s *t* test. Pearson’s test was used for the correlation analysis. A prior sample size calculation was not performed. The Kolmogorov-Smirnov test was used to test the normality of data sets. Data that followed a normal distribution were expressed as mean ± standard error (SEM), and significance was set at 95% confidence.

## RESULTS

### Voluntary physical running improves spatial memory function

**Figure 1.**
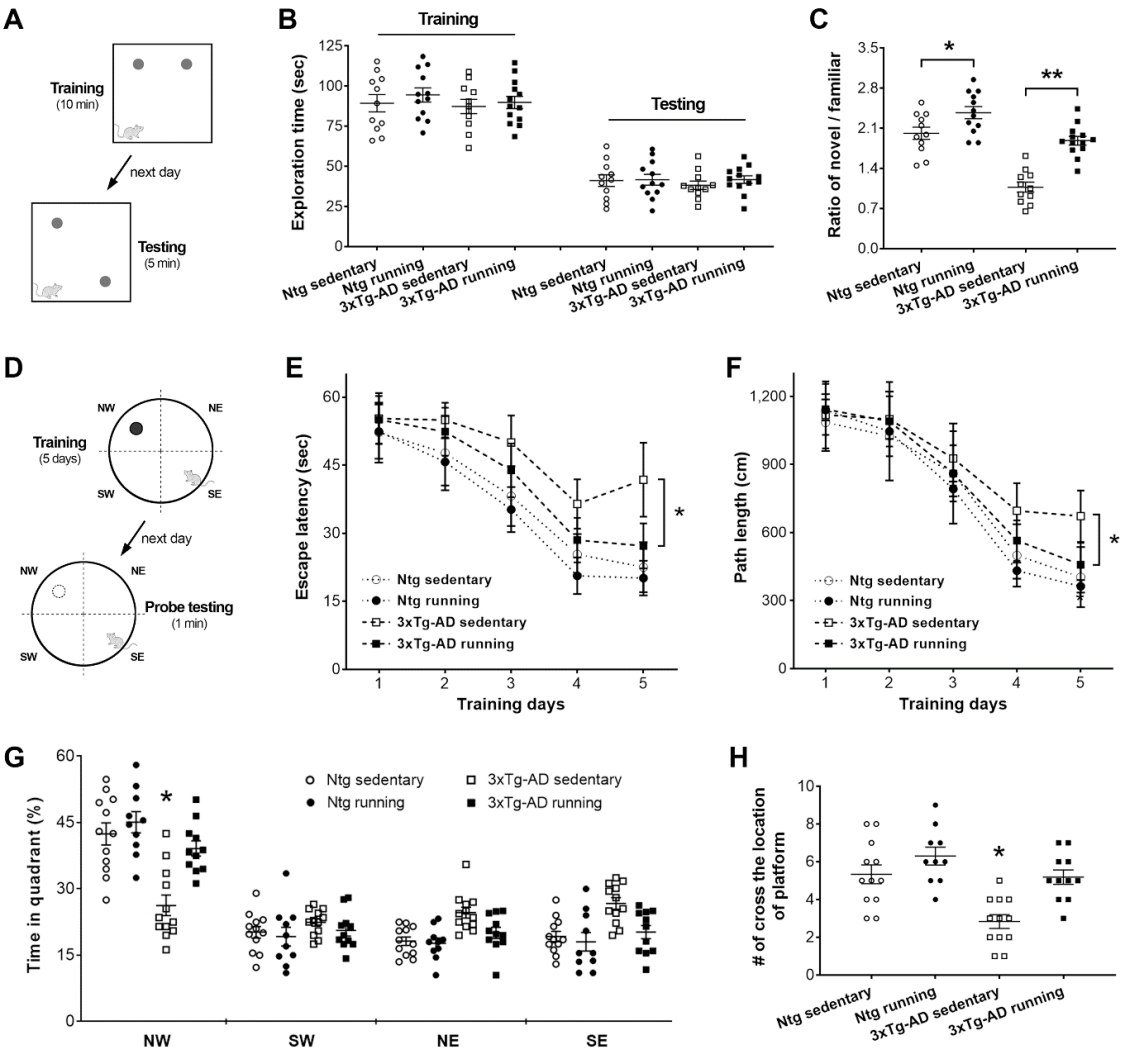
**Voluntary wheel running improves spatial memory**. (**A-C**) Spatial memory was detected via a novel object location task. (**A**) After 5 days of habituation, mice received 10 min of training with two identical objects located at different locations. The testing (5 min) was performed the following day, during which one object was moved to a novel location. The ratio of novel vs. familiar location exploration times was used as an index to represent spatial memory. (**B**) No observed differences in exploration time among the four groups during training (two-way ANOVA, genotype: *F*_[1,43]_ = 0.56, *P*= 0.46; running: *F*_[1,43]_ = 0.73, *P*= 0.40) and testing (genotype × running: *F*_[1,43]_ = 0.22 and 0.46, *P*= 0.64 and 0.50, respectively). (**C**) Runners had a higher novel/familiar ratio compared with controls of the same gene background (Tukey’s *post hoc* test, * *P*< 0.05 and ** *P*< 0.01). n = 11 and 12 mice in Ntg sedentary group and Ntg running group, respectively; n = 11 and 13 mice in 3xTg-AD sedentary and 3xTg-AD running groups, respectively. (D-H) Memory performance in a water maze. (**D**) Mice were trained for 5 days to explore a hidden platform located in the north-west (NW) quadrant, followed by spatial probe testing during which the platform was removed. (E, F) Escape latency and path length to find the platform over training days. On days 4 and 5, 3xTg-AD runners (n = 11) spent less time (two-way ANOVA, day 4: *F*_[1,41]_ = 15.86, *P* = 0.0003; day 5: *F*_[1,41]_ = 25.69, *P*< 0.0001; Tukey’s *post hoc* test, * *P* < 0.05) and swam a shorter distance (day 4: *F*_[1,41]_ = 8.04, *P*= 0.0071; day 5: *F*_[1,41]_ = 14.96, *P*= 0.0004; *post hoc* test, * *P* < 0.05) to reach the hidden-platform compared with 3xTg-AD sedentary mice (n = 12). (G,H) During the probe testing, 3xTg-AD runners and Ntg mice (both runners and controls) preferred exploring the NW platform quadrant more than the other quadrants (three-way ANOVA, *F*_[3,164]_ = 115.9, *P*< 0.0001; *post hoc* test, *P* < 0.05), whereas 3xTg-AD sedentary controls explored all quadrants equally ( *P*> 0.05). In the NW quadrant, 3xTg-AD runners (n = 11) spent more time (in %) (two-way ANOVA, *F*_[1,41]_ = 10.72, *P*= 0.002; *post hoc* test, * *P*< 0.05) and crossed the location of the removed platform more frequently ( *F*_[1,41]_ = 14.64, *P*= 0.0004; *post hoc* test, * *P*< 0.05) versus 3xTg-AD sedentary controls (n = 12).

The effects of voluntary running on spatial memory were investigated in two cohorts of 3xTg-AD mice. The first cohort of mice (Fig. 1A-C) was subjected to a novel object location task that relies largely on an animal's intrinsic preference for novelty and the activity of the dorsal hippocampus [33,42]. Differences were not detected in total exploration time in either the training (two-way ANOVA, *F*_[1,43]_ = 0.73, *P*= 0.40) or testing ( *F*_[1,43]_ = 0.46, *P*= 0.50) phases (Fig. 1B). During the testing (Fig. 1C), 3xTg-AD sedentary controls (7 months old) explored the two objects equally (one-sample *t*-test, *t*_11_ = 0.81, *P* = 0.43) and had declined memory compared to age-matched Ntg sedentary mice (effect of genotype, two-way ANOVA, *F*_[1,43]_ = 57.30, *P*< 0.0001; *post hoc* test, *P*< 0.01), which was consistent with published data [40,43]. However, running wheel for 5 months improved memory in both 3xTg-AD and Ntg mice (effect of running, *F*_[1,43]_ = 38.51, *P*< 0.0001). The novel/familiar ratio of 3xTg-AD runners was significantly higher than that of 3xTg-AD sedentary mice (Tukey’s *post hoc* test, *P*< 0.01). Ntg runners also had an increased novel/familiar ratio versus Ntg sedentary controls ( *P*< 0.05), supporting the beneficial effects of running on spatial memory.

The second cohort of mice (Fig. 1D-H) was subjected to a water maze task. During the 5-day training period, mice required progressively less time (three-way ANOVA, *F*_[4,205]_ = 131.20, *P*< 0.0001) and swam a shorter distance (three-way ANOVA, *F*_[4,205]_ = 216.10, *P*< 0.0001) to find a hidden platform. Differences were not observed in swimming speeds among the groups (two-way ANOVA, *F*_[3,217]_ = 0.94, *P*= 0.42), although the speeds decreased slightly throughout the training period (three-way ANOVA, *F*_[4,205]_ = 21.69, *P*< 0.0001). On days 4 and 5 of training, 3xTg-AD runners spent less time and swam a shorter path to reach the hidden platform compared with 3xTg-AD sedentary mice ( *post hoc* test, * *P* < 0.01), suggesting improved learning and memory ability in 3xTg-AD runners. During the probe testing (Fig. 1G,H), whereas 3xTg-AD sedentary controls spent almost equal time in the four quadrants (one-way ANOVA, *F*_[3,44]_ = 1.61, *P* = 0.20; *post hoc* test, *P*> 0.05), 3xTg-AD runners frequently explored the NW platform quadrant ( *F*_[3,40]_ = 42.63, *P*< 0.0001; *post hoc* test, *P*< 0.05) and spent a higher percentage of time in the platform quadrant than 3xTg-AD sedentary controls (two-way ANOVA, *F*_[1,41]_ = 10.72, *P* = 0.002; *post hoc* test, * *P*< 0.05). Similar to the Ntg mice, the 3xTg-AD runners had higher frequencies of crossing the location of the removed platform than 3xTg-AD sedentary controls (two-way ANOVA, *F*_[1,41]_ = 14.64, *P* = 0.0004; *post hoc* test, * *P*< 0.05). These data indicated that 3xTg-AD runners had improved spatial memory in searching for the original location of the platform.

### An enormous effect of physical running on dendritic spines in 3xTg-AD mice

**Figure 2.**
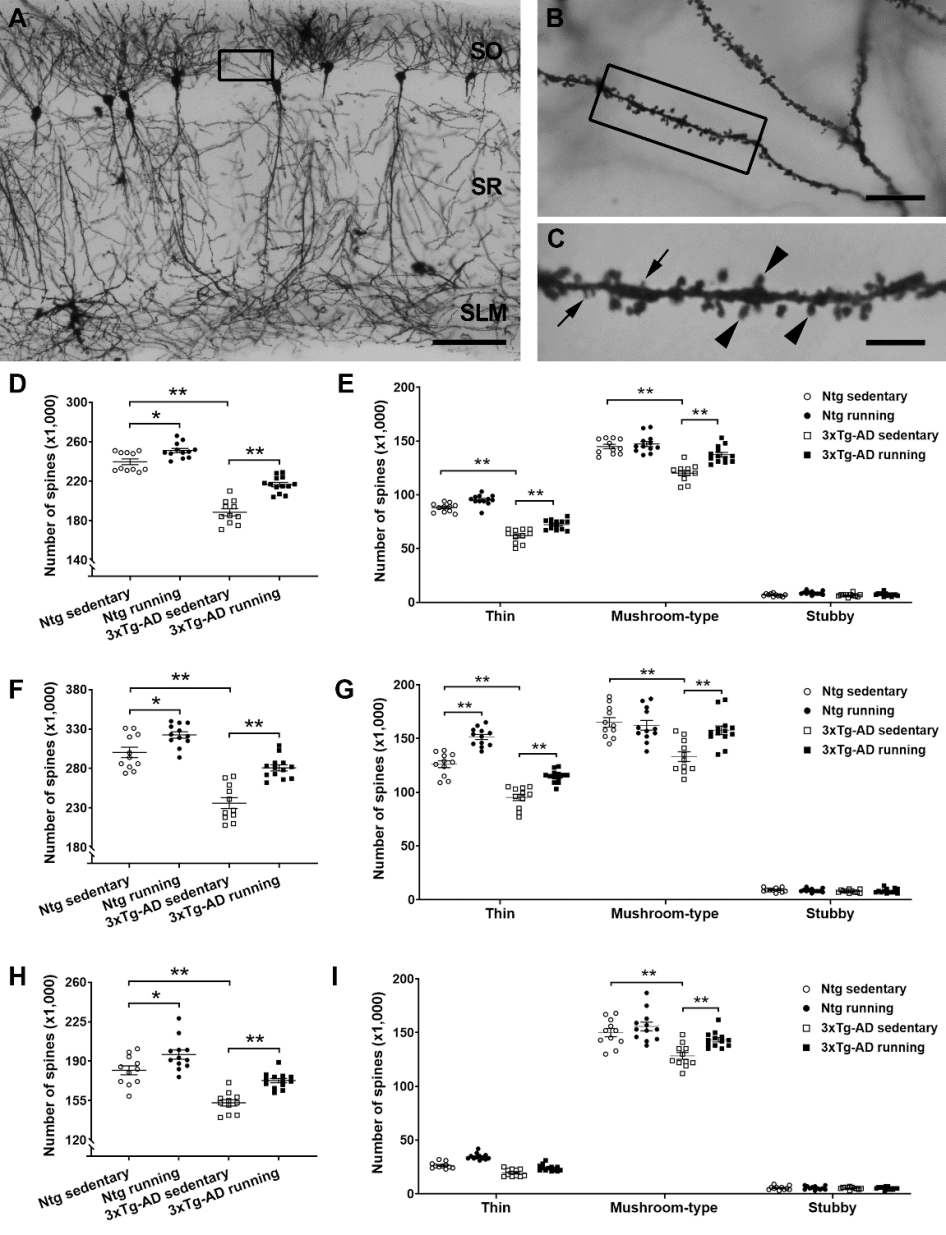
**The effects of physical running on dendritic spines in area CA1 of the dorsal hippocampus**. (**A-C**) Representative images from a 7-month-old 3xTg-AD runner to clarify the location and subcellular domains of Golgi-stained cells in area CA1. Dendritic segments in B were taken from the boxed area in the stratum oriens (SO) in A. The boxed segment in B was further magnified in C to show thin spines (arrows) and mushroom-type spines (arrowheads). Stubby spines were occasionally observed, but not in this image. (**D-I**) Stereological quantitative analyses showed that an increased number of spines was apparent in 3xTg-AD runners (n = 13) and Ntg runners (n = 12) in SO, SR, and SLM when compared with age-matched 3xTg-AD (n = 11) and Ntg sedentary (n = 11) controls, respectively (Tukey’s *post hoc* test, ** *P* < 0.01) (D, F, H). Notably, both thin and mushroom-type spines were protected in the 3xTg-AD running mice compared to 3xTg-AD sedentary controls. However, only thin spines were affected in Ntg-runners compared to Ntg sedentary controls (E, G, I). * *P* < 0.05, ** *P* < 0.01. SR: stratum radiatum; SLM: stratum lacunosum-moleculare. Scale bars: 100 µm in A, 10 µm in B, and 2 µm in C.

**Figure 3.**
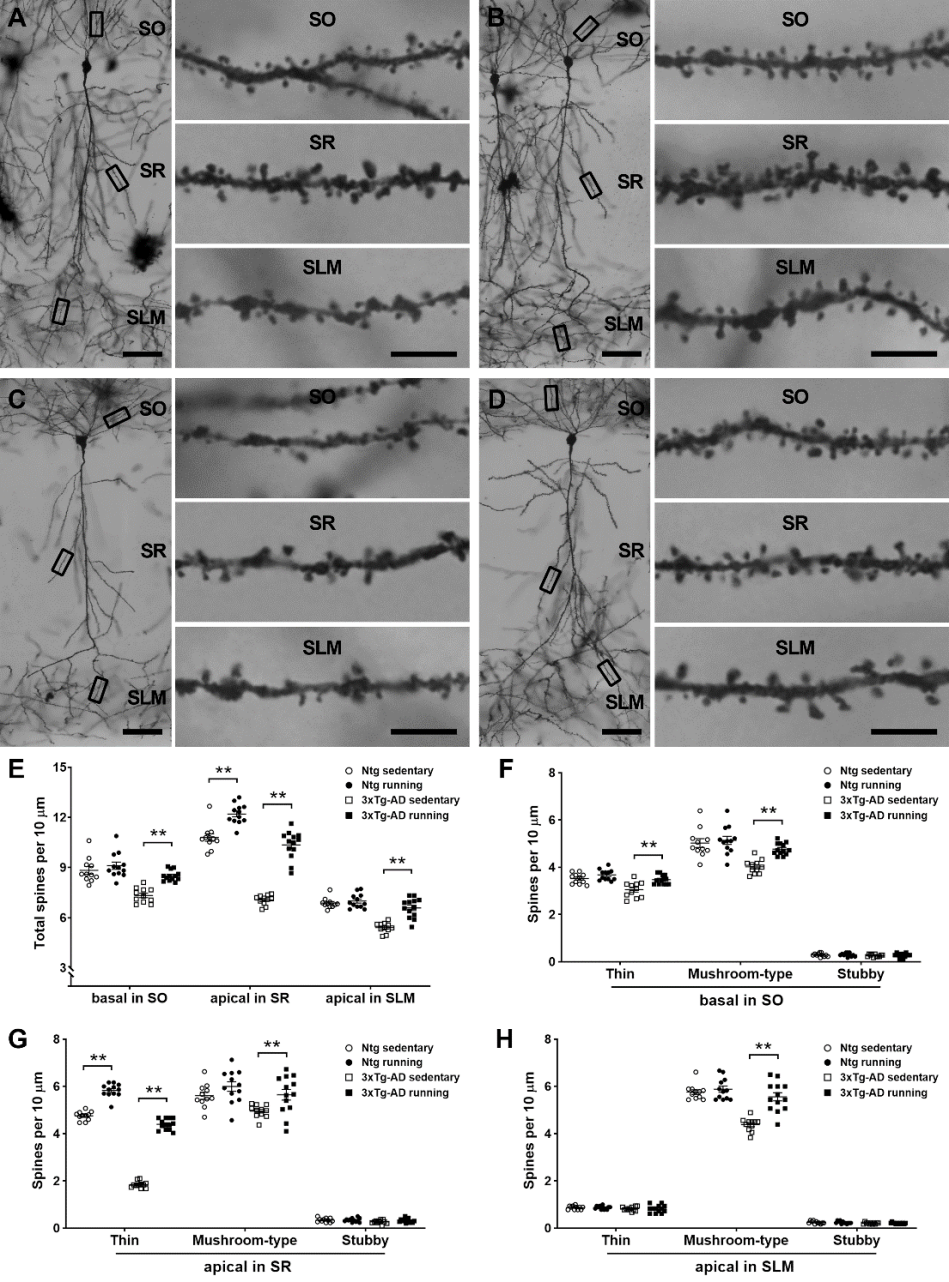
**Physical running protects against the loss of spines on CA1 pyramidal cells in 3xTg-AD mice**. (**A-D**) Representative CA1 pyramidal cells in Ntg sedentary (**A**), Ntg running (**B**), 3xTg-AD sedentary (**C**), and 3xTg-AD running (**D**) mice at 7 months of age. The boxed basal and apical dendritic segments were magnified to show thin and mushroom-type spines. Less spines were observed on basal and apical dendrites in 3xTg-AD sedentary mice (**C**) compared with Ntg sedentary mice (**A**). An increased number of spines was observed in 3xTg-AD runners (**D**) vs. 3xTg-AD sedentary controls (**C**). SO: stratum oriens; SR: stratum radiatum; SLM: stratum lacunosum-moleculare. Scale bars: 50 µm in low magnification images and 5 µm in high magnification images. (**E-H**) Analyses of dendritic spines on individual CA1 pyramidal cells. (**E**) Loss of spines on basal and apical dendrites was apparent in 3xTg-AD sedentary mice vs. Ntg sedentary mice ( *F*_[1,129]_ = 144.8, *P*< 0.0001; *post hoc* test, *P* < 0.01, not marked in the graph). Running affected spines on either basal dendrites or apical dendrites in 3xTg-AD mice, whereas the effect of running was observed on only apical dendrites in Ntg mice (** *P* < 0.01). (**F-H**) Both thin and mushroom-type spines on basal and apical dendrites were protected in 3xTg-AD runners compared with 3xTg-AD sedentary controls (** *P* < 0.01). Only thin spines on apical dendrites in SR were affected by running in Ntg mice. Spine density is expressed as the number of spines per 10 µm dendritic segment. A three-way ANOVA was performed with genotype (Ntg vs. 3xTg-AD), running, and spine type/dendritic branch as factors, followed by Tukey’s or Bonferroni’s *post hoc* test. The values represent the mean ± S.E.M, n = 11 and 12 mice in Ntg sedentary and running groups, respectively; n = 11 and 13 mice in 3xTg-AD sedentary and running groups, respectively.

Our previous studies on aged mice have revealed that running wheel for 5 months can protect against aging-related loss of dendritic spines on CA1 hippocampal cells, which is important for restoring spatial memory function in aging mice [31-33]. We thus focused on area CA1 and explored the effects of running on spines in 3xTg-AD mice when they completed a spatial memory performance test. Dendritic spines were evaluated via two approaches: unbiased stereological counting (Fig. 2) and reconstructed pyramidal cell analysis (Fig. 3).

The spines were first counted in strata oriens (SO), radiatum (SR), and lacunosum-moleculare (SLM) of area CA1 via an unbiased stereological fractionator method (Fig. 2). Two-way ANOVA analyses revealed a running-induced alteration in the number of spines in SO, SR, and SLM ( *F*_[1,43]_ = 52.05, *F*_[1,43]_ = 38.84, *F*_[1,43]_ = 26.36, respectively, all *P*< 0.0001). An increased spine number was apparent in 3xTg-AD runners versus 3xTg-AD sedentary controls (Tukey’s *post hoc* test, ** *P* < 0.01) as well as in Ntg runners versus Ntg controls (* *P* < 0.05). A genotype-related difference in spine numbers was also detected in SO, SR, and SLM ( *F*_[1,43]_ = 244.0, *F*_[1,43]_ = 97.34, *F*_[1,43]_ = 62.85, respectively, all *P*< 0.0001) with lower spine numbers in 3xTg-AD sedentary mice versus Ntg controls (Tukey’s *post hoc* test, ** *P* < 0.01). As spines in the adult hippocampus are classified into three subtypes (thin, mushroom-type, and stubby) that are involved in different learning and memory processes [31,44], we asked whether an increased number of spines in the runners originated from a specific type of spines. Interestingly, 3xTg-AD runners had more thin spines in SO and SR (three-way ANOVA, *F*_[1,129]_ = 53.17 and *F*_[1,129]_ = 43.16, respectively, all *P*< 0.0001; *post hoc* test, ** *P* < 0.01) and more mushroom-type spines in SO, SR, and SLM (** *P* < 0.01), compared with 3xTg-AD sedentary controls. In Ntg runners, there were more thin spines in SO and SR (** *P* < 0.01), but a similar number of mushroom-type spines ( *P* > 0.05), compared with Ntg sedentary controls.

The spines were further analyzed on the basal and apical dendrites of reconstructed individual CA1 pyramidal cells because the distribution of spines is highly branch-dependent (Fig. 3). As expected, a lower density of spines on basal and apical dendrites was apparent in 3xTg-AD sedentary mice versus Ntg sedentary mice (three-way ANOVA, *F*_[1,129]_ = 144.8, *P*< 0.0001; *post hoc* test, ** *P* < 0.01). Running protected against the loss of spines on CA1 pyramidal cells ( *F*_[1,129]_ = 88.64, *P*< 0.0001). Spines on basal and apical dendrites were well preserved in 3xTg-AD runners compared with 3xTg-AD sedentary controls (** *P* < 0.01). Notably, running affected both thin and mushroom-type spines on the basal dendrites ( *F*_[1,129]_ = 10.17, *P*= 0.0018) as well as on apical dendrites (in SR: *F*_[1,129]_ = 58.53, *P*< 0.0001; in SLM: *F*_[1,129]_ = 13.37, *P*= 0.0004. Tukey’s *post hoc* test, ** *P* < 0.01) in 3xTg-AD mice. However, the effect of running in Ntg mice was limited to thin spines on apical dendrites in SR ( *F*_[2,129]_ = 252.5, *P*< 0.0001; *post hoc* test, ** *P* < 0.01) (Fig. 3E). All together, physical running protected against the loss of spines on CA1 pyramidal cells in 3xTg-AD mice, and this effect extensively occurred on thin and mushroom-type spines.

**Figure 4.**
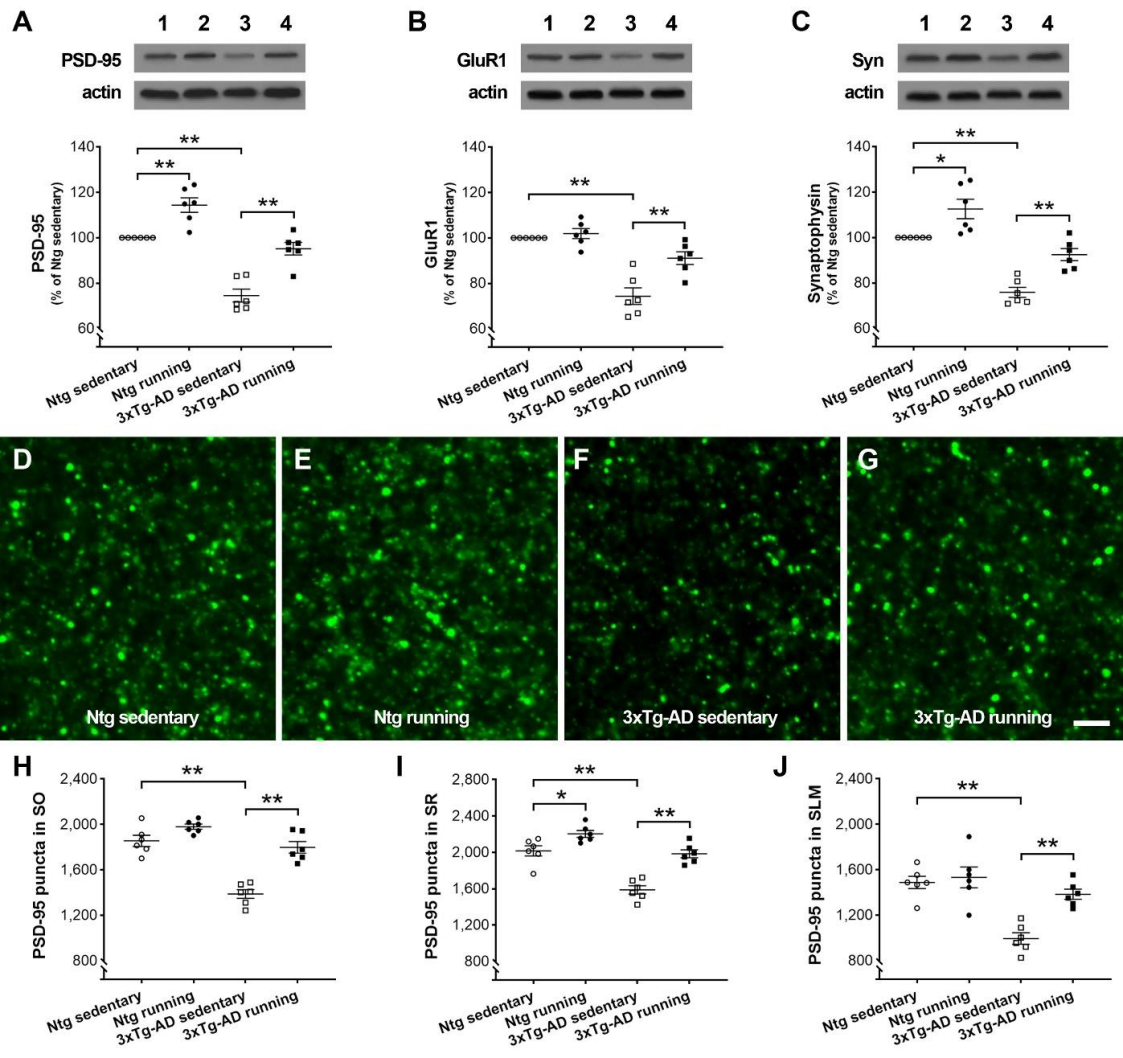
**Physical running alleviates synaptic deficits in the dorsal hippocampus of 3xTg-AD mice**. (**A-C**) The levels of synaptic proteins including PSD-95, GluR1, and synaptophysin in hippocampal CA1. Representative bands from Ntg sedentary (1), Ntg running (2), 3xTg-AD sedentary (3), and 3xTg-AD running (4) mice at 7 months of age. The optical densities of bands corresponding to PSD-95, GluR1, and synaptophysin were normalized to respective protein levels of actin and expressed as % of Ntg sedentary controls. Whereas running wheel for 5 months upregulated the expressions of PSD-95 and synaptophysin, but not GluR1 in Ntg mice, running protected against the loss of all three measured proteins in 3xTg-AD mice (data were expressed as mean ± SEM, ** *P* < 0.01, * *P* < 0.05, n = 6). (**D-J**) Effects of running on PSD-95-ir puncta in area CA1. Representative confocal z-stack images in SR from Ntg sedentary (**D**), Ntg running (**E**), 3xTg-AD sedentary (**F**), and 3xTg-AD running (**G**) mice. Quantitative analysis of PSD-95-ir puncta (**H-J**). Three-way ANOVA analysis (genotype × running × region) showed a significant genotype effect ( *F*_[1,60]_ = 121.2, *P*< 0.0001) and running effect ( *F*_[1,60]_ = 77.64, *P*< 0.0001) with genotype-by-running interaction ( *F*_[1,60]_ = 22.70, *P*< 0.0001). Tukey’s *post hoc* test, ** *P* < 0.01, * *P* < 0.05. n = 6 mice per group. SO: stratum oriens; SR: stratum radiatum; SLM: stratum lacunosum-moleculare. Scale bars: 4 µm in D-G.

### Physical running alleviates synaptic deficits in 3xTg-AD mice

To explore the synaptic basis of the beneficial effects of running on spatial memory and dendritic spines, we measured the expression of synaptic proteins and counted the synapses in the dorsal hippocampus. The protein levels of postsynaptic density protein-95 (PSD-95), AMPA glutamate receptor subunit GluR1, and synaptophysin (the major synaptic vesicle protein p38 located in presynaptic terminal buttons) were measured in area CA1 of 3xTg-AD and Ntg mice for both the running and sedentary groups via Western blot assays (Fig. 4). 3xTg-AD running mice had elevated levels of synaptic proteins compared with 3xTg-AD sedentary controls (two-way ANOVA, *F*_[1,20]_ = 46.85, 13.38, and 28.13 for PSD-95, GluR1, and synaptophysin, respectively; Tukey’s *post hoc* test, ** *P* < 0.01). Ntg runners had higher expressions of PSD-95 (** *P* < 0.01) and synaptophysin (* *P* < 0.05) versus Ntg sedentary controls. As expected, a decreased expression of synaptic proteins was apparent in 3xTg-AD sedentary mice compared with Ntg sedentary mice ( *F*_[1,20]_ = 76.31, 51.07, and 64.84 for PSD-95, GluR1, and synaptophysin, respectively; all *P* < 0.0001. The *post hoc* test, ** *P* < 0.01).

The expression of PSD-95 was further evaluated on immunostaining sections because PSD-95 has been detected on both thin and mushroom-type spines, and PSD-95-immunoreactive (ir) puncta can represent synapses in the hippocampus [33]. PSD-95-ir puncta were stereologically counted in the following subregions of area CA1: SO, SR, and SLM. As shown inFigure 4, loss of PSD-95-ir puncta/synapses was apparent in all measured regions in 3xTg-AD sedentary mice versus Ntg sedentary controls (three-way ANOVA, genotype × running × region, *F*_[1,60]_ = 121.2, *P*< 0.0001; *post hoc* test, ** *P*< 0.01). The 3xTg-AD runners had comparable numbers of synapses in measured regions versus Ntg mice, which were higher than those in 3xTg-AD sedentary controls (three-way ANOVA, *F*_[1,60]_ = 77.64, *P*< 0.0001; *post hoc* test, ** *P* < 0.01). Notably, the effects of running on PSD-95-ir puncta were limited to SR in Ntg mice. Taken together, these data are consistent with the effects of running on dendritic spines in area CA1, supporting the conclusion that prolonged voluntary running protects against a loss of spines and synapses in hippocampal CA1 of 3xTg-AD mice.

**Figure 5.**
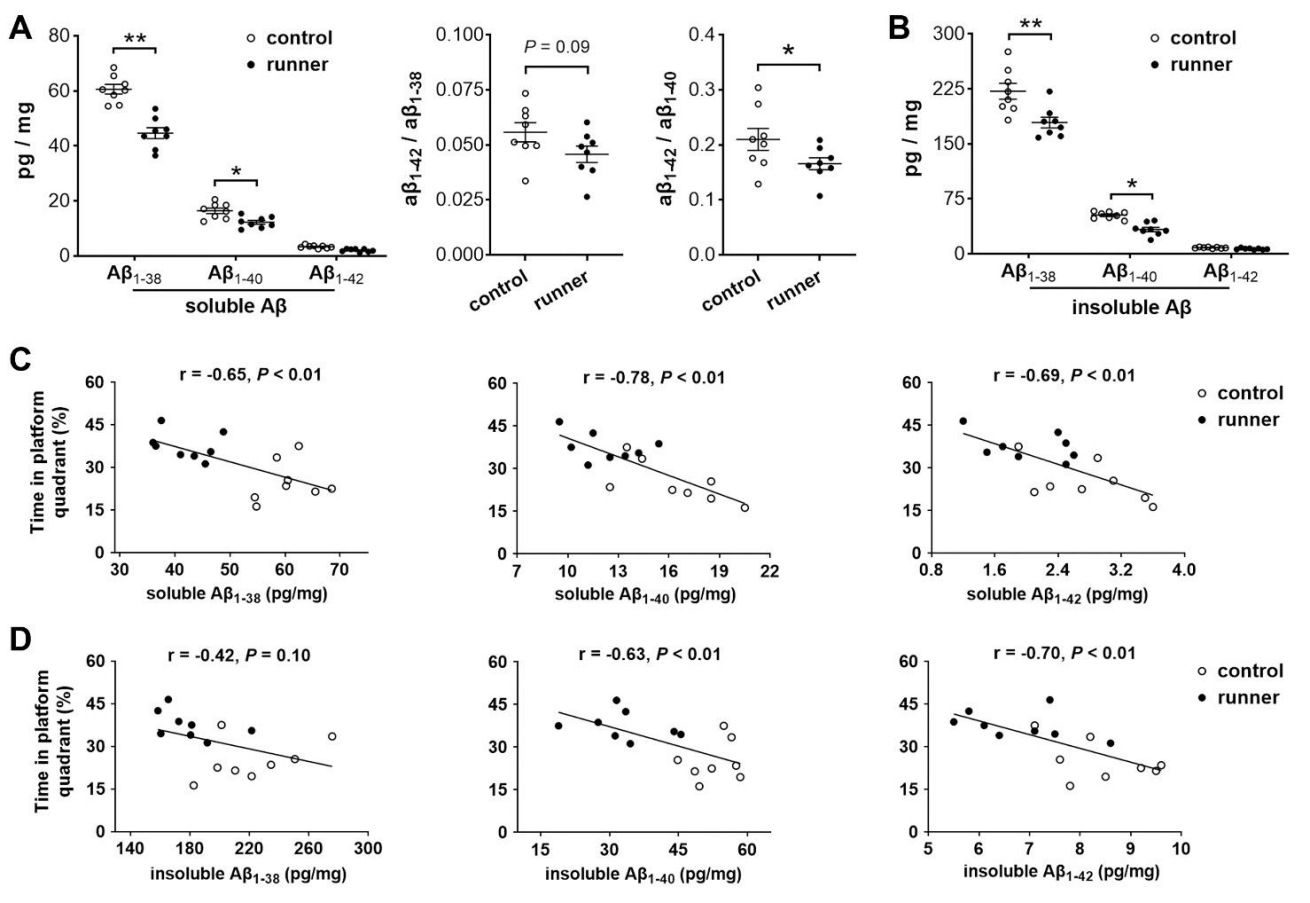
**Physical running reduces the levels of Aβ_1-38_ and Aβ_1-40_ in the dorsal hippocampus, which is beneficial to spatial memory performance in 3xTg-AD mice**. (A, B) Voluntary running for 5 months resulted in decreased levels of Aβ peptides including soluble (**A**) and insoluble (**B**) fractions in area CA1 of the dorsal hippocampus. The running mice (n = 8) had a decreased ratio of soluble Aβ_1-42_/soluble Aβ_1-40_ compared with the sedentary mice (n = 8). * *P* < 0.05, ** *P*< 0.01. (C, D) Correlation between the levels of Aβ peptides and spatial memory performance. Negative correlations were observed between the % time spent in the platform quadrant and soluble Aβ peptides (**C**) (Pearson r = -0.65, -0.78, and -0.69 for Aβ_1-38_, Aβ_1-40_, and Aβ_1-42_, respectively, all *P* < 0.01). The % time in the platform quadrant negatively correlated with insoluble Aβ peptides including Aβ_1-40_ and Aβ_1-42_ (**D**) (r = -0.63 and -0.70, respectively, *P* < 0.01), but not Aβ_1-38_ (r = -0.42, *P* = 0.1).

**Figure 6.**
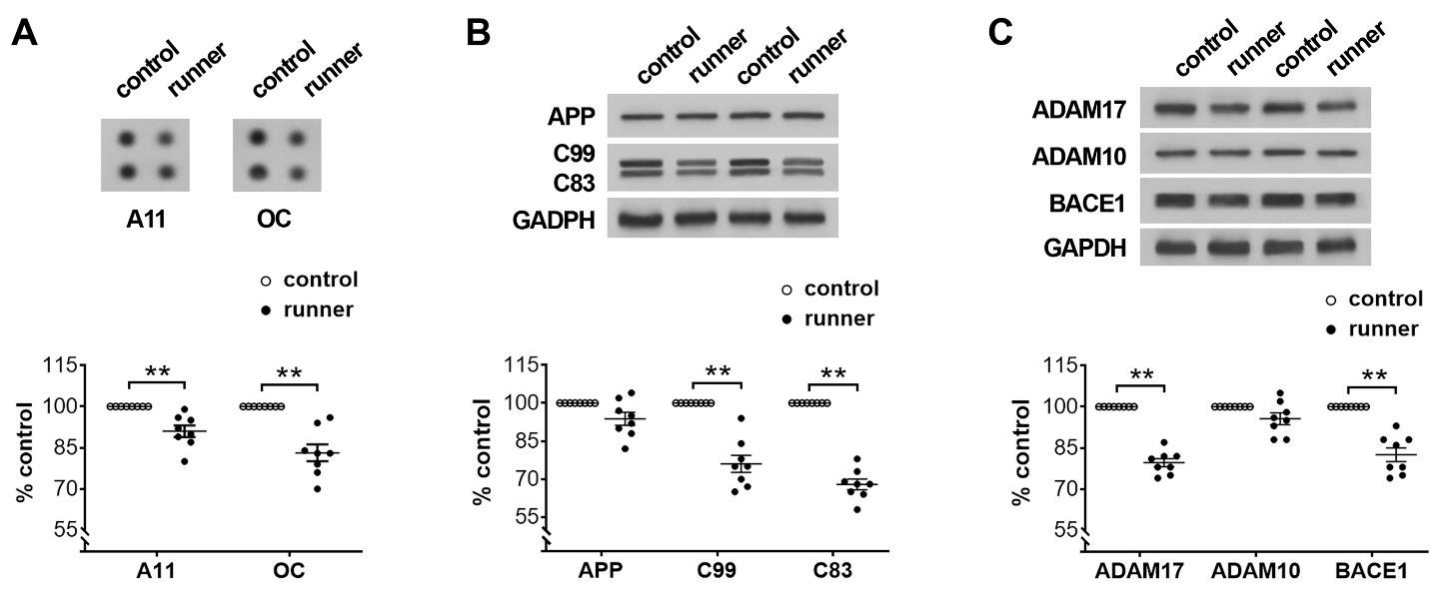
**Prolonged physical running downregulates APP processing in the dorsal hippocampus**. (**A**) Decreased Aβ-oligomers including AB40 oligomers (A11) and amyloid fibril (OC) in area CA1 of the dorsal hippocampus of 3xTg-AD running mice compared to sedentary controls. (**B**) The levels of APP holoprotein and APP C-terminal fragments (C99 and C83) in area CA1. Representative bands from 3xTg-AD sedentary controls and 3xTg-AD runners. The optical densities of bands corresponding to APP, C99, and C83 were normalized to respective protein levels of GAPDH and expressed as % of sedentary controls. No difference in APP, but downregulation in C99 and C83 in 3xTg-AD runners vs. sedentary controls. (**C**) Analyses of α-secretases ADAM-17 and ADAM-10 and β-secretase BACE1 in tissues of area CA1. The optical densities of bands were normalized and expressed as % of controls. ** *P*< 0.01, n = 8 mice per group.

### Physical running reduces the levels of Aβ peptides and oligomers in 3xTg-AD mice

Given the enormous effects of running on spines/synapses and synaptic proteins in 3xTg-AD mice versus Ntg mice, we investigated whether prolonged running had any effects on Aβ peptides and oligomers (Figs. 5, 6). At the end of a water maze testing, a group of 3xTg-AD running mice was compared with 3xTg-AD sedentary mice (n = 8 mice per group). Lower levels of soluble Aβ including Aβ_1-38_ and Aβ_1-40_ were detected in 3xTg-AD runners compared with sedentary controls (two-way ANOVA, running × peptide subtype, *F*_[1,42]_ = 55.62, *P*< 0.0001; *post hoc* test, * *P*< 0.05 for Aβ_1-40_ and ** *P*< 0.01 for Aβ_1-38_). A lower Aβ_1-42_/Aβ_1-40_ ratio was found in the runner group versus control group (* *P* < 0.05) (Fig. 5A). Less insoluble Aβ_1-38_ and Aβ_1-40_ were also measured in 3xTg-AD runners ( *F*_[1,42]_ = 22.50, *P*< 0.0001; *post hoc* test, * *P*< 0.05 for Aβ_1-40_, ** *P*< 0.01 for Aβ_1-38_) (Fig. 5B). Notably, the levels of Aβ peptides correlated with spatial memory performance. Specifically, the percentage of time spent in the platform quadrant negatively correlated with the measured levels of soluble and insoluble Aβ peptides in hippocampal CA1 (Fig. 5C,D).

The effects of running on Aβ oligomers were further investigated. Dot-blot data suggested reduced levels of Aβ oligomers positive for AB40 oligomers (A11) and amyloid fibril (OC) in 3xTg-AD runners ( *F*_[1,28]_ = 48.51, *P*< 0.0001; *post hoc* test, ** *P*< 0.01) (Fig. 6A). To explore whether APP processing was involved in the running-provoked decrease of Aβ peptides and oligomers, full-length APP and APP C-terminal fragments C99 and C83 were measured via Western blot analyses. No difference was observed in steady-state levels of full-length APP holoprotein between 3xTg-AD runners and sedentary controls ( *F*_[1,42]_ = 173.4, *P*< 0.0001; *post hoc* test, *P*= 0.08). However, C99 and C83 were downregulated in the runners ( *F*_[1,42]_ = 173.4, *P*< 0.0001; *post hoc* test, ** *P*< 0.01) (Fig. 6B). Considering that the cleavage of APP requires α-secretase and β-secretase, α-secretases ADAM 10 and ADAM 17 as well as β-site APP cleaving enzyme 1 (BACE1) were analyzed (Fig. 6C). Decreased levels of ADAM 17 and BACE1 were apparent in 3xTg-AD runners compared with sedentary controls ( *F*_[1,56]_ = 103.0, *P*< 0.0001; *post hoc* test, ** *P*< 0.01). Together, these data suggest that the running-provoked decrease of Aβ levels may be caused by the downregulation of APP processing.

**Figure 7.**
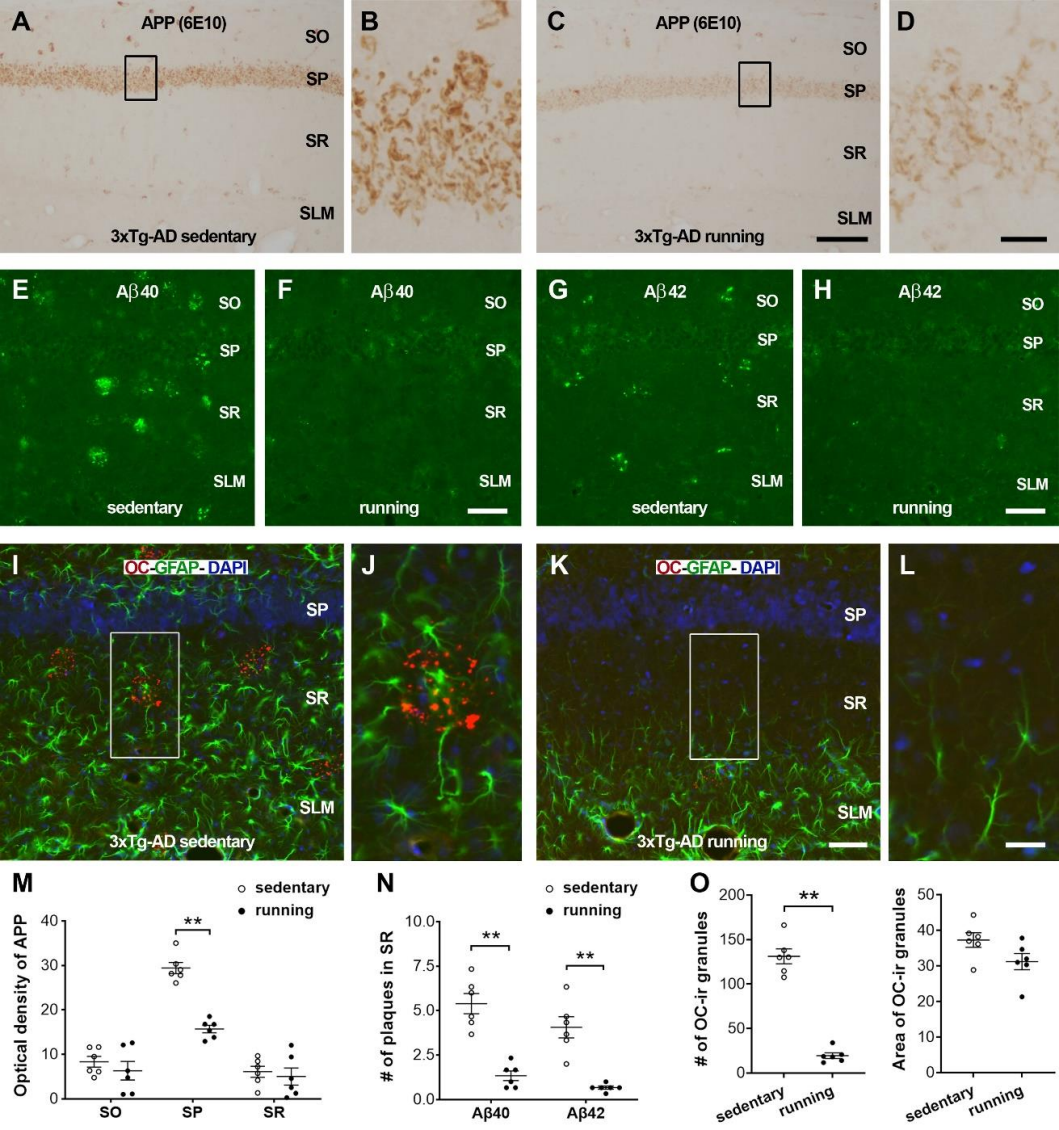
**The changes of Aβ pathology in response to physical running are evaluated on the hippocampal sections**. (**A-D**) Representative images of intracellular APP from 3xTg-AD sedentary (A, B) and running (C, D) mice. APP immunoreactivity was detected primarily in the stratum pyramidale (SP) of area CA1. Boxed areas in A and C were magnified in B and D, respectively to show the intracellular expression of APP in 7-month-old mice. (**E-H**) Aβ40 and Aβ42 aggregates (plaques) were often observed in area CA1 of 3xTg-AD sedentary mice, but occasionally detected in the runners. (**I-L**) Immunostaining of fibrillar oligomers (OC, red) and astrocytes (GFAP, green) in area CA1. Boxed areas in the stratum radiatum (SR) were magnified to display the reduced accumulation of OC-ir clusters and decreased proliferation of astrocytes in response to voluntary physical running. (**M-O**) Quantification. (**M**) The optical density of APP intracellular immunoreactivity in three layers of area CA1 ( *F*_[1,30]_ = 25.21, ** *P*< 0.01). (**N**) The number (per 450 µm × 200 µm) of extracellular Aβ40 and Aβ42 plaques in CA1 radiatum. Fewer plaques observed in runners vs. controls ( *F*_[1,20]_ = 73.47, ** *P*< 0.01). (**O**) The number (per 450 µm × 200 µm) and area of individual OC-ir granules in CA1 radiatum. Reduced number of OC-ir granules observed in runners vs. controls ( *t*_10_ = 12.44, ** *P* < 0.01). Data presented as mean ± SEM, n = 6 mice per group. SO: stratum oriens; SLM: stratum lacunosum-moleculare. Scale bars: 100 µm in A, C; 15 µm in B, D; 50 µm in E-H, I, K; and 20 µm in J, L.

Moreover, the expressions of APP, Aβ40, Aβ42, and Aβ-oligomer were evaluated immunohistochemically on the hippocampal sections of 3xTg-AD runners and sedentary controls (Fig. 7). Intracellular APP detected via 6E10 (Fig. 7A-D) was found primarily in the stratum pyramidale of area CA1, which is consistent with previous reports [43,45]. APP immunoreactivity was observed less frequently in strata oriens and radiatum. A decreased OD value of APP immunoreactivity was identified in the pyramidale (two-way ANOVA, *F*_[1,30]_ = 25.21, *P*<0.0001; Bonferroni’s *post hoc* test, ** *P*< 0.01), but not in the oriens and radiatum ( *post hoc* test, *P*= 0.91 and 0.99, respectively) of runners when compared with controls (Fig. 7M). Extracellular Aβ deposits (plaques) recognized by Aβ40 and Aβ42 antibodies (Fig. 7E-H) were often observed in the CA1 radiatum of 3xTg-AD sedentary controls. In this layer, fewer Aβ40 and Aβ42 plaques were observed in runners versus controls ( *F*_[1,20]_ = 73.47, *P*<0.0001; Bonferroni’s *post hoc* test, ** *P*< 0.01 for both Aβ40 and Aβ42) (Fig. 7N). The fibrillar oligomers were revealed by using anti-amyloid fibrils OC (Fig. 7I-L). In 3xTg-AD sedentary mice, dual labeling of OC and GFAP revealed that the accumulation of OC-ir clusters and proliferation of astrocytes were obvious in the radiatum where loss of spines/synapses was apparent. The number of OC-ir clusters was higher in controls versus runners (4.33 ± 0.82 vs. 0.83 ± 0.75; *t*_10_ = 7.72, *P* < 0.01). The number of individual OC-ir granules inside the clusters was reduced in response to physical running ( *t*_10_ = 12.44, ** *P* < 0.01). No difference was found on the average area per granule between the two groups ( *t*_10_ = 1.99, *P* = 0.07) (Fig. 7O). These immuno-histochemical data further corroborate the beneficial effects of prolonged voluntary running on Aβ pathology in the dorsal hippocampus.

## DISCUSSION

Physical exercise has a wide spectrum of impacts on brain structure that are beneficial to memory health. Here we observe the effect of prolonged wheel running on the defective spatial memory of a triple-transgenic mouse model of AD (3xTg-AD mouse) and investigate its underlying mechanisms. The major findings are as follows. (i) Voluntary running improves hippocampal-dependent spatial memory in 3xTg-AD mice. (ii) Daily running (1 hour per day) for 5 months prevents loss of dendritic spines on CA1 pyramidal cells in 3xTg-AD mice. The beneficial effects of running on spines are accompanied by increased individual synapses and enhanced expressions of synaptic proteins PSD-95, GluR1, and synaptophysin in area CA1. (iii) The effects of running on spines are enormous in 3xTg-AD mice compared with Ntg mice, as both thin and mushroom-type spines are preserved in 3xTg-AD mice, while only thin spines are preserved in Ntg mice. (iv) Running at a moderate pace reduces the levels of Aβ peptides and oligomers via downregulating APP processing. Particularly, reduced accumulation of Aβ peptides/ oligomers is apparent in the radiatum of area CA1, and running-induced enhancement of memory performance correlates with a reduced level of Aβ peptides in the dorsal hippocampus.

Physical exercise can improve cognitive function and reduce the risk of AD [ *e.g.*, 46-48]. Patients with AD dementia show improvements in memory and cognitive behavior when they start exercising regularly [8-15,18,49]. This study provides evidence for the beneficial effect of voluntary running on spatial memory and its underlying mechanisms in 3xTg-AD mice. Because the 3xTg-AD mice display cognitive impairments by 6 months of age [43,50] in a sex-dependent manner [51-53], male mice are employed in the study and all memory performances are tested at 7 months old. During the training days of a water maze memory task, 3xTg-AD mice spend more time and swim in a longer path to find the platform on the last two days of training compared with Ntg mice, suggesting that 3xTg-AD mice have impaired learning and memory, consistent with previous reports [40,43]. Running for 5 months improves spatial memory in both 3xTg-AD mice and non-transgenic wild-type mice, supported by data from a probe trial in which the platform is removed. During this trial, the 3xTg-AD runners crossed the location of the removed platform more frequently than sedentary controls did, and they also spent a larger percentage of time in the platform quadrant. Furthermore, data from the object location task imply that memory decline is apparent in 3xTg-AD sedentary mice compared with Ntg controls, confirming the declined memory observed in the water maze. Similarly, 3xTg-AD running mice have improved memory, indicated by a higher ratio of novel to familiar location exploration compared to Ntg sedentary controls, further supporting the beneficial effects of physical running on spatial memory.

How could physical running help prevent loss of spatial memory in 3xTg-AD mice? Spatial memory largely relies on the integrity of structural components of synaptic connections in area CA1 of the dorsal hippocampus. In area CA1, pyramidal cells harbor numerous spines, representing the main target of excitatory synaptic inputs including the Schaffer axon terminals and entorhinal fibers [54,55]. These pathways are highly associated with hippocampal-dependent spatial navigation [56,57]. Generally, defeat of postsynaptic targets may result in degeneration of presynaptic elements, leading to a loss of synaptic connections. Therefore, the conservation of dendritic spines in area CA1 will benefit synaptic connections and spatial memory. Our studies on normal aging mice have shown that physical running initiated at an older age and lasted for 5 months enhances hippocampal-dependent memory function in the mice. Particularly, enhanced memory performance correlates with an increased density of dendritic spines in area CA1 of old running mice [31,32]. Here, the number and types of spines are analyzed on CA1 pyramidal cells. Focusing on thin and mushroom-type spines, a significant loss of both thin and mushroom-type spines is found in 3xTg-AD sedentary mice compared with age-matched non-transgenic mice. However, 3xTg-AD mice subjected to 5 months of running have a higher density of spines compared with age-matched sedentary controls. Interestingly, 3xTg-AD running mice have increased counts of both thin and mushroom-type spines on CA1 pyramidal cells, distinctive from the selective effect of running on thin spines in Ntg mice. The limited effect of running observed in Ntg mice is consistent with our reports on aged C57 mice, which found that 5 months of running protects against the age-related loss of thin spines and small synapses in the hippocampus [31,33]. An increased number of spines in area CA1 provides more chances for forming synaptic contacts in this area, a basis of enhanced synaptic plasticity and memory function. Further studies are needed to understand the mechanisms responsible for the profound effect of running on thin and mushroom-type spines in the hippocampus of 3xTg-AD mice.

The beneficial effects of physical running on dendritic spines in 3xTg-AD mice are accompanied by a measurable change of synapses and enhanced expression of synaptic proteins. The analysis of PSD-95-ir puncta in area CA1 suggests increased synapses in 3xTg-AD running mice, supporting the effects of running on dendritic spines, because PSD-95 is detected on both mushroom-type and thin spines [33,58]. Increased PSD-95-ir puncta in all examined subfields of 3xTg-AD running mice further reveals the profound effects of long-term voluntary running in 3xTg-AD mice. The protein levels of PSD-95 and GluR1 measured by Western blotting are consistent with the analyses of thin and mushroom-type spines. Increased expression of GluR1 has been detected in 3xTg-AD running mice versus 3xTg-AD sedentary controls, suggesting the preservation of mushroom-type spines and big synapses. This is because AMPA receptors are rarely detected on thin spines and small synapses [59,60], and GluR1-ir puncta have been used as a synaptic marker to represent mushroom-type spines [33]. In the current study, we have further examined the expression of synaptophysin, a major synaptic vesicle protein located in the presynaptic terminals [32]. Synaptophysin is found in all types of synapses and is important for synaptic integrity [61,62]. Synaptophysin immunoreactivity completely matches with the synaptic profile distribution, and increased expression of synaptophysin is accompanied by the formation of synapses [61]. Previous studies from patients with AD have suggested that synaptophysin is more vulnerable than other synaptic proteins such as syntaxin or SNAP-25, and loss of synaptophysin is an early event that correlates with initial cognitive impairment [63-65]. Consistent with the loss of postsynaptic targets, reduced expression of synaptophysin is apparent in area CA1 of 3xTg-AD sedentary mice. Interestingly, daily voluntary running for 5 months prevents the loss of synaptophysin in the 3xTg-AD mice, which may contribute to the protective effect of running on memory function as indicated by the correlation analyses in our previous aging study [32]. Taken together, these data suggest that prolonged voluntary running prevents the loss of pre- and post-synaptic proteins in 3xTg-AD mice.

It is interesting to note that dendritic spines and synaptic proteins are largely protected in 3xTg-AD runners compared to non-transgenic runners. Because soluble Aβ is critical to the early stage of synaptic failure seen in AD pathogenesis [66] and intracellular Aβ pathogenesis precedes the appearance of tau pathology in the 3xTg-AD mice [43,45], we investigated the potential effects of physical running on Aβ pathology in area CA1. To understand the specific roles of different types of Aβ peptides at the early stage of synaptic alteration in response to voluntary physical running, we analyzed the soluble and insoluble Aβ peptides as well as Aβ oligomers via ELISA and Dot-blotting. An increased level of Aβ in the brain is an early and specific phenomenon associated with the progression of AD. Specifically, Aβ oligomers can bind to synaptic sites and lead to a loss of spines in organotypic hippocampal slice cultures [37,67]. The current study finds that wheel running for 5 months decreases the levels of both soluble and insoluble Aβ peptides as well as the formation of Aβ-oligomers. In the 3xTg-AD runners, both soluble and insoluble Aβ_1-38_ and Aβ_1-40_ are significantly decreased compared with sedentary controls. The levels of Aβ peptides including soluble and insoluble fragments negatively correlate with spatial memory performance, indicating the importance of Aβ peptides in hippocampal synaptic plasticity and memory function [67,68]. Particularly, a decreased ratio of soluble Aβ_1-42_ versus soluble Aβ_1-40_ is detected in 3xTg-AD runners compared with their sedentary controls, supporting the note that the Aβ_1-42_/Aβ_1-40_ ratio could serve as a marker of imminent onset or progression of AD [69,70].

It is known that Aβ is generated from β-amyloid precursor protein (APP) through sequential proteolytic cleavages by a group of secretases. Here, the levels of full-length APP and its C-terminal fragments in area CA1 are detected via Western blotting by using a specific anti-APP-CT20 antibody [40,71]. In the hippocampus, the expression of APP is cellular and laminal dependent [71]. APP and its fragments can influence synaptic function at both pre- and post-synaptic sites. The overexpression of APP in either dendritic or axonal compartments may lead to reduced spine density and synaptic plasticity in nearby neurons [72]. We find that running downregulates APP C-terminal fragments C99 and C83 as well as α-secretases ADAM 17 and β-secretase BACE1 in 3xTg-AD mice, suggesting that running-provoked downregulation of Aβ pathogenesis occurs in part by modulating APP processing. Immunostaining further reveals that prolonged running reduces the accumulation of both intracellular APP and extracellular Aβ peptides/oligomers in area CA1. Importantly, diminished Aβ plaques and fibrillar oligomers are obvious in the striatum radiatum, where the beneficial effects of running on dendritic spines and synapses occur. Additional research should be carried out to identify the causal link between spine collapse and amyloid beta accumulation. Considering the important role of microglia in the maturation of dendritic spines and the stabilization of synaptic contacts [8], the effects of physical running on microglial phagocytosis of spines/synapses are worth investigating.

In summary, prolonged voluntary running improves hippocampal dependent spatial memory and reduces the accumulation of Aβ peptides and oligomers in 3xTg-AD mice. The enormous effects of running on dendritic spines and synaptic proteins in hippocampal area CA1 in the AD model mice compared with non-transgenic mice indicate that voluntary running may protect dendritic spines and synapses by reducing amyloid beta levels. These data suggest that prolonged running improves spatial memory in preclinical AD via slowing down amyloid pathology and preventing loss of synaptic contacts.
